# Editorial: Neuromuscular diagnostics and sensorimotor performance in training and therapy - beyond the pure biomechanical approach

**DOI:** 10.3389/fspor.2023.1296276

**Published:** 2023-10-09

**Authors:** Heiner Baur, Benoit Pairot De Fontenay, Susan Sigward

**Affiliations:** ^1^ School of Health Professions, Division of Physiotherapy, Bern University of Applied Sciences, Bern, Switzerland; ^2^ Inter-University Laboratory of Human Movement Science, Université Claude Bernard, Lyon, France; ^3^ Division of Biokinesiology and Physical Therapy, University of Southern California, Los Angeles, CA, United States

**Keywords:** neuromuscular control, functional adaptation, sensorimotor training (SMT), training adaptation, therapy, neuromechanical adaptation, biopsychosocial (BPS) model, biomedical model

**Editorial on the Research Topic** Neuromuscular diagnostics and sensorimotor performance in training and therapy - beyond the pure biomechanical approach

Traditional movement science research has adopted methodology that describes differences in movement among groups or conditions using biomechanical variables to infer underlying features of neuromuscular control. Historically, this approach marked the onset of the complex analysis of movement biomechanics bring relevant insights into the mechanics of human movement (1).

Hypotheses were developed by extrapolating data from healthy active to injured populations (2). The last decade before the millennium provided first references to the neuromechanical aspects of movement, thus expanding the view towards the neuromotor control aspect of movement generation and adjustment (3). In the 2000s, substantial evidence is reported on spinal and supraspinal adaptations after balance or sensorimotor training which delivered important evidence-based knowledge that was rapidly implemented in clinical practice (4). Moreover, studies that combined both “mechanical” and “neuromuscular” views evolved (5, 6). We can postulate, that we still need more evidence-based knowledge on the interplay between the underlying neurophysiologic movement generation and the observed mechanical motor output. These integral neuro-biomechanical approaches still rely heavily on a biomedical perspective that is lately challenged by the call for biopsychosocial paradigms to cover all relevant aspects in human movement analysis to draw meaningful conclusions for diagnostics, prevention and therapy (7).

Research can rarely incorporate all dimensions at one time but our claim should be that we focus on experimental paradigms that purposely integrate both biomechanical and neuromechanical pieces of the puzzle to seek a more comprehensive understanding of typical and impaired movement. There are promising examples of such approaches that now combine classic biomechanical research with neurophysiological methods and patient reported outcomes or other psychometric measures (8, 9).

The aim of this Research Topic is therefore to provide a collection of studies that contribute to these integrative approaches by using diverse viewpoints and subsequently diverse methodology from study protocols, scoping or systematic reviews or experimental and interventional studies. They all contribute with different pieces of the puzzle “beyond the pure biomechanical approach”.

Three investigations provided insight into motor control and muscle coordination in patient populations and those with experimentally imposed pian. Bartsch-Jimenez et al. described differences in “fine synergies” derived from electromyographic data of multiple lower leg muscles between persons with foot drop and controls that may reflect potentially relevant for motor adaptations to impaired ankle control. Chan and Sigward found that achieving loading symmetry in standing requires attention in those who are recovering from ACL reconstruction while it is more automatic in healthy controls. Bertrand-Charette et al. described the influence of acute ankle pain on motor output and performance of a standard balance test used to assess function in individuals with ankle injuries. While these studies targeted specific adaptations, Quarmby et al.’s systematic review of evidence regarding mechanical and neuromuscular control impairments in individuals with Achilles tendinopathy highlights limited consensus and areas for future work.

Other contributors provided insight into the effects of neurocognitive and neurophysiological based interventions. Rogan and Taeymans describe in their systematic review the evidence of positive effects of whole-body vibration on sensorimotor function in the elderly which highlights the therapeutic potential in this population. Faes et al. investigated the effects of a whole-body vibration intervention on several dimensions like movement control, well-being, and cognition in a randomized controlled trial. Hegi et al. summarized the existing body of evidence on sensor-based augmented visual feedback that should be used in coordination training to elicit sensorimotor adaptations. Mourits et al. describe a study protocol of a quasi-randomized controlled trial investigation of a game based intervention that combines neurocognitive effects of an external focus of attention and game like motivation along with patient specific real time spine motion to improve movement control of the spine. Finally, Mathieu-Kälin et al. described an assessment tool for develop to measure movement quality during hop tests. This tool adds important valuation of the control strategies used to complete a task beyond that of just performance.

The goal of the Research Topic was accomplished by presenting studies that incorporated a variety of manscirpt that represent “out of the box” neuro-biomechanical approaches to investigate underlying features of impaired movement. The broad range of paradigms and methodological approaches of the Research Topic certainly reflects the initial idea and the contributions highlight different aspects on the pathway to more mutifaceted approaches.

The guest editor team would love to see many views, downloads, and citations of the papers included in this Research Topic and we anticipate that in the future more contributions to Frontiers and Sports and Active living could be “virtually” added to this topic.
